# A novel *LGI1* mutation causing autosomal dominant lateral temporal lobe epilepsy confirmed by a precise knock‐in mouse model

**DOI:** 10.1111/cns.13761

**Published:** 2021-11-12

**Authors:** Ping Hu, Dan Wu, Yan‐Yu Zang, Yan Wang, Ya‐Ping Zhou, Fengchang Qiao, Xiao‐Yu Teng, Jiang Chen, Qing‐Qing Li, Jia‐Hui Sun, TingTing Liu, Hao‐Yang Feng, Qi‐Gang Zhou, Yun Stone Shi, Zhengfeng Xu

**Affiliations:** ^1^ Department of Prenatal Diagnosis Women's Hospital of Nanjing Medical University Nanjing Maternity and Child Health care Hospital Nanjing China; ^2^ Minister of Education Key Laboratory of Model Animal for Disease Study Model Animal Research Center Department of Neurology Drum Tower Hospital Medical School Nanjing University Nanjing China; ^3^ State Key Laboratory of Pharmaceutical Biotechnology National Resource for Mutant Mice Jiangsu Key Laboratory of Molecular Medicine Medical School Nanjing University Nanjing China; ^4^ School of Pharmacy Nanjing Medical University Nanjing China; ^5^ Chemistry and Biomedicine Innovation Center Nanjing University Nanjing China; ^6^ Institute for Brain Sciences Nanjing University Nanjing China

**Keywords:** ADLTE, antiepileptic drugs, knock‐in mouse model, *LGI1* mutation, protein secretion

## Abstract

**Aims:**

This study aimed to explore the pathomechanism of a mutation on the leucine‐rich glioma inactivated 1 gene (*LGI1*) identified in a family having autosomal dominant lateral temporal lobe epilepsy (ADLTE), using a precise knock‐in mouse model.

**Methods and Results:**

A novel *LGI1* mutation, c.152A>G; p. Asp51Gly, was identified by whole exome sequencing in a Chinese family with ADLTE. The pathomechanism of the mutation was explored by generating *Lgi1^D51G^* knock‐in mice that precisely phenocopied the epileptic symptoms of human patients. The *Lgi1^D51G^* ^/^ *^D51G^* mice showed spontaneous recurrent generalized seizures and premature death. The *Lgi1^D51G^* ^/+^ mice had partial epilepsy, with half of them displaying epileptiform discharges on electroencephalography. They also showed enhanced sensitivity to the convulsant agent pentylenetetrazole. Mechanistically, the secretion of Lgi1 was impaired in the brain of the D51G knock‐in mice and the protein level was drastically reduced. Moreover, the antiepileptic drugs, carbamazepine, oxcarbazepine, and sodium valproate, could prolong the survival time of *Lgi1^D51G^* ^/^ *^D51G^* mice, and oxcarbazepine appeared to be the most effective.

**Conclusions:**

We identified a novel epilepsy‐causing mutation of *LGI1* in humans. The *Lgi1^D51G^* ^/+^ mouse model, precisely phenocopying epileptic symptoms of human patients, could be a useful tool in future studies on the pathogenesis and potential therapies for epilepsy.

## Graphical abstract

*Lgi1* ^D51G^ knock‐in mice (c.152A>G; p. Asp51Gly) quite precisely phenocopied the epileptic symptoms of the human patients in a novel Chinese ADLTE family and would be a useful tool for future study on the pathogenesis and potential therapy for epilepsy.

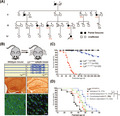

## 1 INTRODUCTION

As the name suggests, autosomal dominant lateral temporal lobe epilepsy (ADLTE) (OMIM 600512) is an epileptic syndrome originating in the lateral temporal lobe and follows an autosomal‐dominant pattern of inheritance. It is characterized by partial seizures and is usually accompanied by prominent auditory or aphasic symptoms.^1^, ^2^, ^3^, ^4^, ^5^ The pathomechanism in up to 50% of the families with this syndrome has been attributed to the heterozygous mutations of the leucine‐rich glioma inactivated 1 (*LGI1*) gene located on chromosome 10q23.23.^6^, ^7^ To date, over 40 *LGI1* mutations have been reported in familial ADLTE, including 29 missense mutations distributed along with the LGI1 protein.^8^, ^9^, ^10^ *Lgi1* knockout mice and transgenic mice expressing mutant *Lgi1* in a knockout background showed the epileptic phenotype.^8^, ^11^ Homozygous *Lgi1* knockout mice (*Lgi1* ^−/−^) displayed spontaneous recurrent generalized seizures with clonic and tonic limb convulsions and died prematurely. Partial *Lgi1* deletion *(Lgi1* ^+/−^ *)* and transgenic mice expressing mutant *Lgi1* were more susceptible to convulsant agent pentylenetetrazole (PTZ) and acoustic stimuli than wild‐type animals.^8^, ^11^, ^12^

Lgi1 is a secretory protein expressed predominantly in neurons performing various important functions.^13^, ^14^ It has been reported that Lgi1 inhibits the inactivation of the presynaptic voltage‐gated potassium channel Kv1 by interacting with the cytoplasmic Kvβ subunit.^15^ The secreted Lgi1 assembles into dimers at the synaptic cleft. Here, one Lgi1 molecule binds to the presynaptic A disintegrin and metalloprotease 23 (ADAM23), while the other molecule couples with the postsynaptic α‐amino‐3‐hydroxy‐5‐methyl‐4‐isoxazolepropionic acid‐type glutamate receptor (AMPAR) through the postsynaptic ADAM22 linked to the postsynaptic density protein 95 platform, thus regulating AMPAR‐mediated synaptic transmission trans‐synaptically.^9^, ^11^, ^16^, ^17^ Lgi1, Adam22, Adam23, and Kv1 knockout mice show similar lethal epileptic phenotypes.^8^, ^11^, ^12^, ^18^, ^19^, ^20^, ^21^ Recent studies also indicate that Lgi1 modulates the intrinsic excitability of pyramidal neurons in the lateral temporal lobe cortex, whose dysfunction contributes to the epileptic phenotype.^22^, ^23^ Lgi1 may regulate neuronal development, such as the pruning of glutamatergic synapses in the hippocampus, neuronal growth, and development of the cortex and cerebellum.^24^, ^25^, ^26^, ^27^ Furthermore, Lgi1 appears to regulate myelination.^28^, ^29^

In this study, we identified a novel *LGI1* mutation, wherein aspartic acid (D) was substituted with glycine (G) at position 51 (D51G), in a Chinese family with ADLTE. The residual Asp51 is evolutionarily conserved throughout the species. We explored the pathomechanism of the mutation by generating *Lgi1^D51G^* knock‐in mice. Both *Lgi1^D51G^* ^/^ *^D51G^* and *Lgi1^D51G^* ^/+^ mice displayed the epileptic phenotype. Biochemical assays indicated that the secretion of the Lgi1 was defective in *Lgi1^D51G^* ^/^ *^D51G^* mice. Thus, the epileptic phenotype in the family with ADLTE with heterozygous *LGI1^D51G^* mutation most likely arises from the defective secretion of LGI1 protein. Additionally, we tested the effects of the commonly used antiepileptic drugs, including carbamazepine, oxcarbazepine, and sodium valproate, on *Lgi1^D51G^* ^/^ *^D51G^* mice. All the drugs prolonged the survival of the mice, but oxcarbazepine appeared to be the most effective.

## 2 MATERIALS AND METHODS

### 2.1 Human participants and data collection

This study was approved by the Medicine Ethics Committee of Nanjing Maternity and Child Health Care Hospital (2019 KY‐081). All participants were interviewed personally, and they provided informed consent. Patient information regarding seizure semiology, age at seizure onset, neurologic examinations such as electroencephalography (EEG) and magnetic resonance imaging, and treatment outcomes were collected.

### 2.2 Genetic analysis of human participants

Genome samples were collected from all the available family members. Whole exome sequencing (WES) using an Agilent V6 capture chip with 100X sequencing depth was conducted in five affected members (II‐9, III‐5, III‐7, III‐11, and IV‐6) and two unaffected members (II‐6 and III‐1). The *LGI1* fragment of four affected members (II‐9, III‐5, III‐7, and III‐11) and seven unaffected members (II‐3, II‐6, III‐1, III‐3, III‐12, III‐16, and IV‐4) were amplified and subsequently confirmed by Sanger sequencing.

### 2.3 Generation of the *Lgi1^D51G^* knock‐in mice

All animal‐related research followed the Animal Research: Reporting of In‐vivo Experiments 2.0 guidelines^30^ and was approved by the Institutional Animal Care and Use Committee of Model Animal Research Center at Nanjing University. The *Lgi1^D51G^* knock‐in mice were generated using the CRISPR‐Cas9 strategy at GemPharmatech (Nanjing, China). Cas9 messenger RNA (mRNA), single‐guide RNA (sgRNA), and donor vectors carrying the fragment with the mutant site were co‐injected into zygotes. The sgRNA targeting sequence was 5′‐TTGCTACAAGTACACACGGC‐3′. The sgRNA guided Cas9 endonuclease to the target sequence and cleaved the DNA at both strands, which generated a double‐strand break (DSB). The DSB was repaired by homologous recombination with donor vectors, resulting in the substitution of “D” with “G” at position 51 of the Lgi1 protein sequence. The founders were crossed with C57BL/6 mice to generate *Lgi1^D51G^* ^/+^ mice. Given that the *Lgi1^D51G^* ^/^ *^D51G^* mice showed premature death, we crossed the heterozygous mice to obtain homozygous mice. Genotypes were determined by sequencing the fragments amplified using the following primers: 5′‐GACCTGTTCTTAGAGCAAGACAATC‐3′ and 5′‐TGTTAGTGCTGTCAAATGGTCAGG‐3′. The sequencing primer was 5′‐GACCTGTTCTTAGAG CAAGACAATC‐3′.

### 2.4 Primary culture of cortical neurons

The cortical neurons were acutely dissected and cultured following the protocol detailed in a previous study.^31^ Briefly, the neurons were prepared from the cortex of wild‐type *Lgi1^D51G^* ^/+^ and *Lgi1^D51G^* ^/^ *^D51G^* mice at E18.5 and plated at a density of 5 × 10^5^ cells/cm^2^ in 6‐cm dishes. The cells were cultured in Neurobasal‐A medium containing 2% B27, 1% L‐glutamine, and 1% penicillin‐streptomycin (all from Gibco) and collected for analysis at DIV4.

### 2.5 EEG monitoring

One week before the EEG recording, 2‐month‐old wild‐type and *Lgi1^D51G^* ^/+^ mice were anesthetized by isoflurane inhalation during electrode implantation. For hippocampal recordings, bipolar‐twisted stainless‐steel electrodes (PlasticOne) were placed bilaterally in the hippocampi (2.75 mm posterior to the bregma, 2.48 mm lateral to the midline, and 2.88 mm below the dura). Stainless‐steel screws (PlasticOne) were placed epidurally and bilaterally over the frontal cortices (0.5 mm posterior to the bregma and 2.48 mm lateral to the midline). An additional screw was placed just to the right of the frontal sinus and served as the reference electrode. The electrodes were then connected to a connector pedestal (PlasticOne), which was fixed to the skull with dental cement. The mice were left unrestrained for 24–72 h to allow for recovery from surgery before further manipulation, and prolonged EEG monitoring was initiated. Digital EEG recordings were performed using EEG2100 software. Continuous EEG recordings were conducted for each mouse for 4–7 days. Generalized seizures were defined by repetitive epileptiform spiking activity (>3 Hz) that persisted in all electrodes for >3 s. During the recording, the mice were unrestrained in the monitoring boxes, with free access to food and water.

### 2.6 PTZ‐Induced Seizures and Scoring

Seizures were measured in *Lgi1* ^+/+^and *Lgi1^D51G^* ^/+^ mice between postnatal days 21–24 (P21–24). PTZ was injected intraperitoneally at a dose of 40 mg/kg. The seizure‐related activities were examined and scored by an investigator who was blinded to the genotypes of the mice throughout the experiments. Seizures were scored for 10 min after the PTZ injections. The scoring criteria were as follows: 0, no reaction; 1, twitching of the ear, face, and head; 2, myoclonic body jerks; 3, partial clonic convulsion of the forelimbs; 4, generalized clonic convulsions (including four limbs and tail); and 5, generalized tonic convulsions (tonic hindlimb extension or death).^11^, ^32^

### 2.7 Western blotting

The cerebral cortex and primary cultured cortical neurons of the mice were lysed in a radioimmunoprecipitation assay (RIPA) buffer containing 150 mM NaCl, 50 mM Tris Base (pH 8.0), 1% Nondier P‐40, 0.5% Na‐deoxycholate, 1 mM phenylmethylsulfonyl fluoride solution, and complete ethylenediaminetetraacetic acid‐free protease inhibitor cocktail tablet (Roche) with a homogenizer. Lysates were kept on ice for 30 min, followed by centrifugation for 30 min at 12,000 rpm at 4℃, and the supernatants were then collected. For primary cultured neurons, the collected media were centrifuged using Centricon YM30 concentrators (Merck Millipore). The concentrations of the protein samples were measured using the bicinchoninic acid protein assay kit (Genstar). The samples were adjusted to the same protein concentration with 5x loading buffer, RIPA buffer, and dithiothreitol (20 μM for final concentration), which were boiled together at 95℃ for 5 min before being separated by 10% sodium dodecyl‐sulfate polyacrylamide gel electrophoresis. Proteins were immunoblotted onto polyvinylidene fluoride membranes (EMD Millipore), which were blocked in 5% nonfat milk dissolved in 150 mM NaCl, 10 mM Tris‐Base (pH 7.4), and 0.1% Tween 20 at room temperature for 1 h. The membranes were then incubated with anti‐Lgi1 antibody (Abcam, ab30868, 1:1000), anti‐glyceraldehyde 3‐phosphate dehydrogenase (GAPDH) (Bioworld, MB001, 1:10000), and horseradish peroxidase‐conjugated secondary antibodies (Bioworld). Protein detection was performed using enhanced chemiluminescence substrates (Tanon, 180–501), and signals were captured using a chemiluminescent imaging system (Tanon). To quantify protein expression, the integrated optical densities of protein bands were measured using ImageJ software (National Institutes of Health); GAPDH was used as the reference.

### 2.8 Real‐time quantitative polymerase chain reaction

Total RNA samples from the cerebral cortex of the mice were extracted using TRIzol (Life Technology) and reverse transcribed into complementary DNA (cDNA) using HiScript Q RT SuperMix for quantitative polymerase chain reaction (qPCR) (+gDNA wiper) kit (Vazyme Biotech, R123). The cDNAs were quantified using the Applied Biosystems StepOnePlus Real‐Time PCR system (Life Technologies) using AceQ^®^ qPCR SYBR^®^ Green Master (High ROX Premixed) (Vazyme Biotech, Q141). Real‐time PCR conditions were 95℃ for 5 min, followed by 40 cycles of 95℃ for 10 s and 60℃ for 30 s. A melt cure of each pair of primers to the target gene was also performed from 60℃ to 95℃, with readings performed every 0.3℃, to ensure the specific reaction. *Gapdh* was used as the reference gene. Expression changes were normalized to wild‐type control. The primers used were as follows: *Lgi1*, 5′‐TTCCTTAACAAATGTGGAC‐3′, and 5′‐TGGGACTTTGCAAATTCTG‐3′ and *Gapdh*, 5′‐TGAACGGGAA GCTCACTGG‐3′, and 5′‐TCCACCACCCTG TTGCTGTA‐3′.

### 2.9 Immunohistochemistry analysis

The mice at P24 were anesthetized and transcranially perfused with 50 ml of 0.01 M phosphate buffer saline (PBS, pH 7.3), and samples were then fixed with 4% paraformaldehyde (pH 7.3). The brains were then collected and subsequently immersed in 0.1 M PBS containing 30% sucrose at 4℃. Transverse brain segments with a thickness of 30 µm were sliced on a cryostat (Leica CM1800, Heidelberg, Germany). For immunohistochemical analysis, the brain slices were pretreated with 3% H_2_O_2_ to exclude endogenous peroxidase. The slices were then blocked in 10% normal donkey serum for 30 min at room temperature and incubated with rabbit anti‐Lgi1 (Abcam, ab30868, 1:100) at 4℃ for 72 h. After incubation with biotin‐conjugated secondary antibody, the slices were stained using avidin‐biotin‐peroxidase system (ABC kit, Maixin) and DAB substrate kit (Vector Laboratories). Images were obtained using Olympus microscope (BX53, Tokyo, Japan).

For immunofluorescence analysis, the brain slices were blocked in 10% normal donkey serum and incubated with anti‐Lgi1 (Abcam, ab30868, 1:100) primary antibody, followed by Alexa Fluor 488‐conjugated (Invitrogen, 1:500) secondary antibody for 4 h at room temperature. The nuclei were labeled with 4′,6‐diamidino‐2‐phenylindole (Life Technologies). A confocal laser microscope (LSM880, ZEISS, Germany) was used to obtain the digital images. Approximately 15 slices obtained from three mice (five slices per mouse) were randomly chosen. The images were captured by individuals blinded to the experimental groups.

### 2.10 Administration of the antiepileptic drugs

Carbamazepine (Sigma, C8981; 66.7 mg/kg), oxcarbazepine (Sigma, O3764; 150 mg/kg), or sodium valproate (Sigma, P4543; 50 mg/kg) was added to the drinking water of the mother mice 14 days after giving birth (P14). After weaning, the pups were housed individually and administered antiepileptic drugs through their drinking water. Survival curves were obtained by Kaplan‐Meier estimates using the log‐rank test.

### 2.11 Statistical analysis

All data are presented as means ± standard errors of mean of at least three independent experiments. Statistical analyses were performed using the GraphPad Prism software (version 8.0). Normality of the data was assessed using the Shapiro‐Wilk test. When normality was achieved, one‐way analysis of variance (ANOVA), followed by Tukey's multiple comparisons test, or unpaired *t* test was used. In the absence of normality, nonparametric Kruskal–Wallis one‐way ANOVA, followed by Dunn's multiple comparisons test, or Mann‐Whitney *U* test was performed. Survival curves of *Lgi1^D51G^* ^/^ *^D51G^* mutant mice treated with antiepileptic drugs were analyzed by Kaplan‐Meier estimates using the log‐rank test. *p*‐values <0.05 were considered statistically significant, and those ≥0.05 were denoted as “ns,” that is, non‐significant.

## 3 RESULTS

### 3.1 Participants and clinical descriptions

**FIGURE 1:**
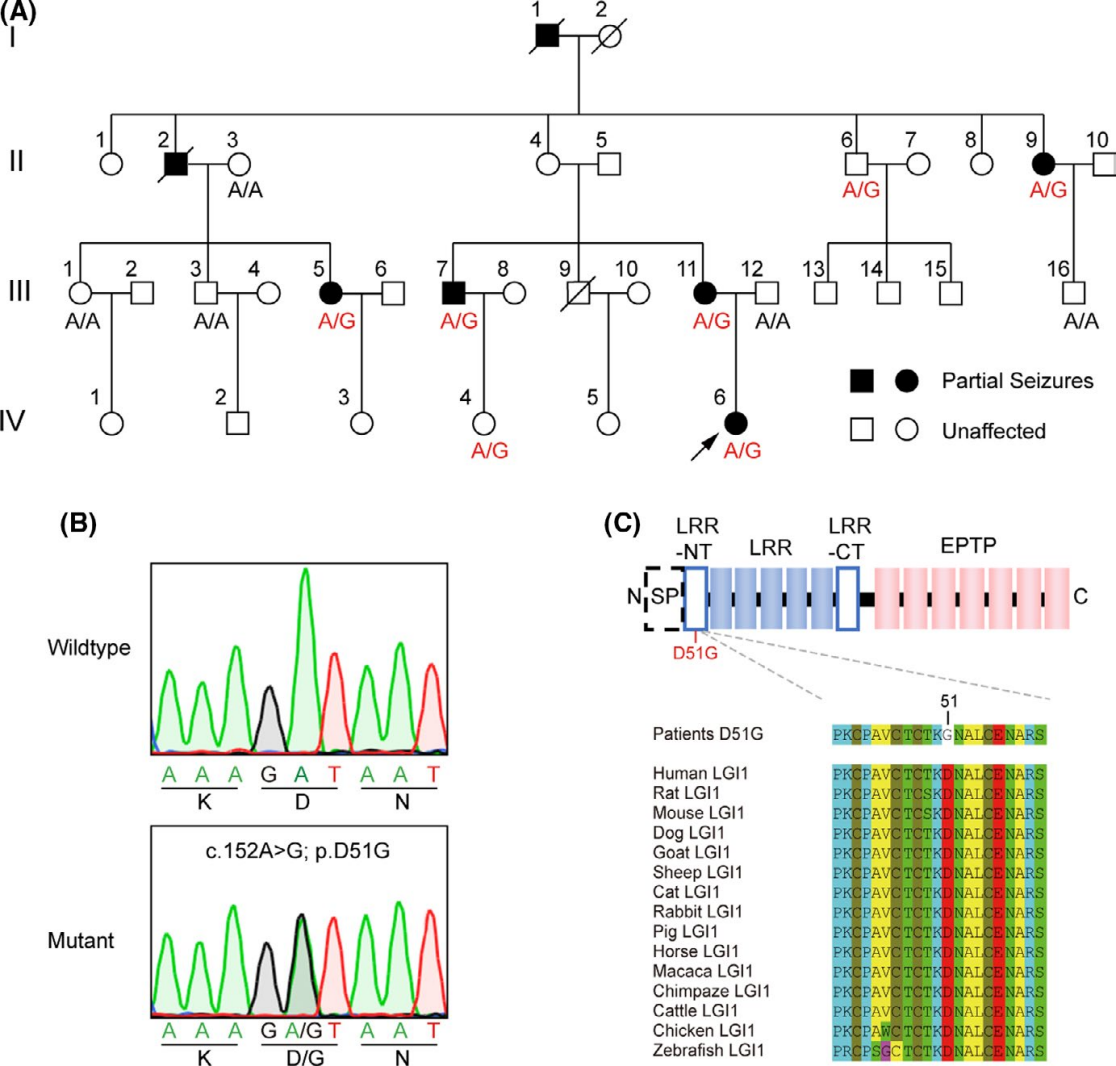
Family pedigree and the mutation. (A) Pedigree of a four‐generation family with seizures. Circle, female; square, male; symbol with diagonal line, deceased family member; blackened symbol, subject with symptom; black arrow marked the proband. Sequence results of all available members were indicated. Individuals carrying heterozygous c.152A>G mutation were marked by A/G; those without mutation were marked by A/A. (B) Sanger sequencing traces of individuals with and without c.152A>G mutation. (C) Sequence alignment of human LGI1 and other species. The domain organizations of LGI1 were displayed above the alignment. The mutation site was marked in red at N‐terminal of LRR. LRR, leucine‐rich repeat; EPTP, epitempin repeat; NT, N‐terminal; CT, C‐terminal; SP, signal peptide

The proband (IV‐6) was a Chinese girl who experienced her first seizure at the age of 12 years. Her epileptic fits generally lasted for 2–3 min, presenting as sudden fall, convulsion of the limbs, unconsciousness, eyes turning white, foaming at the mouth, trismus, and tongue bite. Based on her clinical manifestations and EEG results, the condition was diagnosed as generalized tonic‐clonic seizures. She was treated effectively with carbamazepine and had a family history of seizures (Figure 1A). Among all her family members, seven experienced partial seizures: one in the first generation (I‐1), two of six in the second generation (II‐2 and II‐9), three of ten in the third generation (III‐5, III‐7, and III‐11), and one of six in the fourth generation (IV‐6, the proband) (Figure 1A). The other family members did not report any seizures.

### 3.2 Genetic analysis of the family with ADLTE

The inheritance showing an autosomal dominant pattern prompted us to conduct WES for the members of this family. WES in five affected family members (II‐9, III‐5, III‐7, III‐11, and IV‐6) revealed a heterozygous c.152A>G mutation in exon 1 of the *LGI1* gene, resulting in the substitution of “D” with “G” at position 51 of the protein sequence (p. D51G). The mutation was also detected in an unaffected family member (II‐6), but not in the control (III‐1) (Figure 1A). Sanger sequencing of *LGI1* exon 1 in family members with available blood samples revealed the same heterozygous point mutation in the affected members (II‐9, III‐5, III‐7, III‐11, and IV‐6) but not in the unaffected ones (II‐3, III‐1, III‐3, III‐12, and III‐16). The mutation was also found in the other two asymptomatic individuals (II‐6 and IV‐4), which was in accordance with the incomplete penetrance of *LGI1* mutations (Figure 1A,B).^33^ These results indicated that the family showed characteristic ADLTE. Interestingly, the “D” at position 51, located within the N‐terminus of the leucine‐rich repeat (LRR) domain, is evolutionally conserved throughout the species (Figure 1C).

### 3.3 D51G mutation of Lgi1 causes epileptic phenotype in mice

**FIGURE 2:**
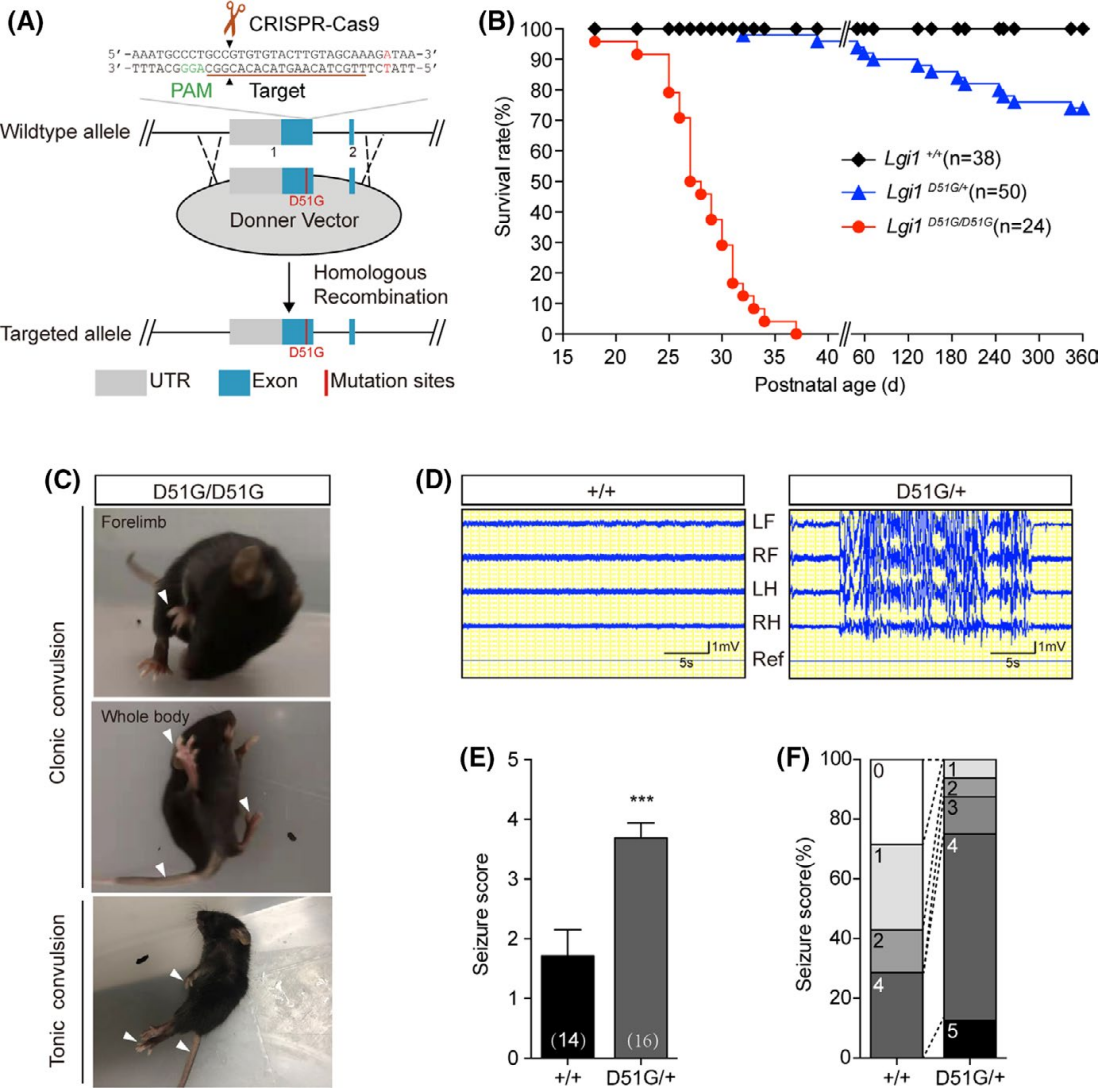
D51G mutation of Lgi1 causes epileptic phenotype in mice. (A) Generation of *Lgi1^D51G^* knock‐in mice by CRISPR‐Cas9 technique. Schematic representation of the wild‐type *Lgi1* allele displayed the target site for sgRNA, Cas9 and donner vector. The sgRNA targeting sequence was labeled in brown. The PAM sequence was labeled in green. The Cas9 cleavage sites were indicated by black arrowhead. (B) Kaplan‐ Meier survival plots of wild‐type (*n* = 38), *Lgi1^D51G^* ^/+^ (*n* = 50) and *Lgi1^D51G^* ^/*D51G*^ (*n* = 24) mice. (C) The epileptic behaviors of *Lgi1^D51G^* ^/*D51G*^ mice at around 3–4 weeks. *Lgi1^D51G^* ^/*D51G*^ mice displayed the spontaneous recurrent seizures with clonic convulsion of forelimbs (upper) and wholebody (middle), followed by full tonic extension (lower). Arrows indicate the limb and tail convulsion. (D) EEG recordings from wild‐type (*n* = 3) and *Lgi1^D51G^* ^/+^ (*n* = 6) mice at 2‐month. LF, left frontal; RF, right frontal; LH, left hippocampus; RH, right hippocampus; Ref, reference. (E) Average seizure scores of wild‐type (1.7 ± 0.4%, *n* = 14) and *Lgi1^D51G^* ^/+^ (3.7 ± 0.3%, *n* = 16) mice to PTZ injection at P21‐24. Number of mice we examined was indicated on the graph. Data are shown as mean ± SEM. ***p < 0.001; unpaired t test. (F) Quantification of reactions to PTZ injection. Criteria were as follows: 0, no reaction; 1, twitching (of ear, face and head); 2, myoclonic body jerks; 3, partial clonic convulsion (of forelimbs); 4, generalized clonic convulsion (including four limbs and tail); 5, generalized tonic convulsions (tonic hindlimb extension, or death)

The substitution of the negatively charged “D” residue with the smallest “G” at this position may hamper the function of LGI1 proteins, resulting in the epileptic phenotype. To verify that *LGI1^D51G^* is the pathogenic cause of ADLTE, we generated *Lgi1^D51G^* knock‐in mice using the CRISPR‐Cas9 strategy (Figure 2A).^34^, ^35^ Both heterozygous (*Lgi1^D51G^* ^/+^) and homozygous (*Lgi1^D51G^* ^/^ *^D51G^*) mice were born in agreement with classic Mendelian laws and without apparent anatomical defects. The *Lgi1^D51G^* ^/^ *^D51G^* mice displayed spontaneous recurrent generalized seizures, with wild jumping and running, followed by clonic and tonic convulsions (Figure 2C, Video S1) at postnatal 3–4 weeks. Sudden noise often triggered seizures in these mice, suggesting sensitivity to sound stimulation. They began to die around P18, and no mice survived for more than 35 days.

Given that ADLTE patients are heterozygous for the D51G allele, we investigated seizure behavior in mice with a heterozygous D51G mutation. We did not observe obvious spontaneous seizures in young *Lgi1^D51G^* ^/+^ mice. However, these mice died earlier than the wild‐type mice did, with the earliest death occurring at around 1 month, and approximately 1/4th of the mice (13/50) died within 1 year (Figure 2B). Early death indicates that they might have experienced spontaneous seizures. Therefore, we conducted EEG monitoring in these mice. Continuous EEG recordings for 1 week of the cerebral cortex and hippocampus at the age of 2 months showed spontaneous epileptiform discharges in half of the *Lgi1^D51G^* ^/+^ mice (3/6), but not in the wild‐type mice (*n* = 3; Figure 2D). These observations were in accordance with the incomplete penetrance of human *LGI1* mutations.^33^ Additionally, the seizure susceptibility of *Lgi1^D51G^* ^/+^ mice in response to PTZ (40 mg/mL), a γ‐aminobutyric acid type A receptor blocker,^36^ was more than that of the wild‐type mice (Figure 2E,F). Thus, the D51G mutation of *Lgi1* causes an epileptic phenotype in mice. *Lgi1^D51G^* ^/^ *^D51G^* and *Lgi1^D51G^* ^/+^ mice are valid models for investigating the pathogenesis of human ADLTE caused by *LGI1^D51G^* mutation.

### 3.4 Secretion of Lgi1 protein is impaired in *Lgi1^D51G^* knock‐in mice

**FIGURE 3:**
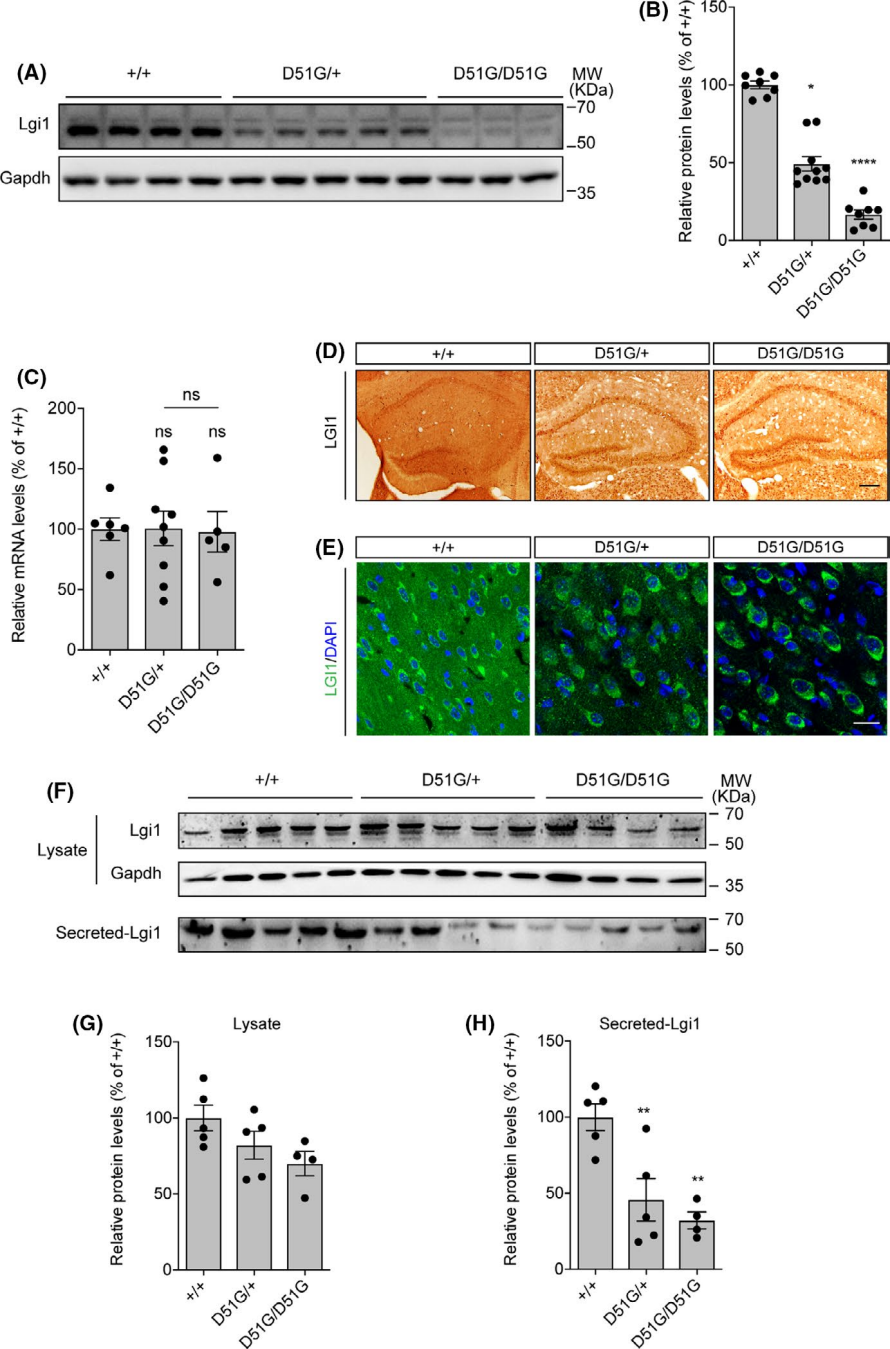
Lgi1^D51G^ protein is unstable and secretion‐defective in mouse brain. (A) Western blot of cortex lysates from wild‐type, *Lgi1^D51G^* ^/+^ and *Lgi1^D51G^* ^/*D51G*^ mice at P22 with anti‐Lgi1 antibody. Gapdh was used as reference. (B) Quantification of Lgi1 protein expression in mouse cortices from two experiments. Lgi1 expression levels relative to Gapdh were normalized to that in wild‐type mice. wild‐type, 100.0 ± 2.4%, *n* = 8; *Lgi1^D51G^* ^/+^, 49.3 ± 4.7%, *n* = 10; *Lgi1^D51G^* ^/*D51G*^, 16.6 ± 3.0%, *n* = 8. Data are shown as mean ± SEM. *p < 0.05; ****p < 0.0001 Nonparametric Kruskal–Wallis one‐way ANOVA followed by Dunn's multiple comparisons test. (C) Relative mRNA expression levels of Lgi1 in cortices of wild‐type (100.0 ± 9.5% *n* = 6), *Lgi1^D51G^* ^/+^ (100.7 ± 14.3%, *n* = 9) and *Lgi1^D51G^* ^/*D51G*^ (97.8 ± 16.9%, *n* = 5). *Gapdh* was used as the reference. Data are shown as mean ± SEM. ns, not significant. One‐way ANOVA followed by Tukey's multiple comparisons test. (D) Immunohistochemical analysis of Lgi1 in hippocampus of wild‐type, *Lgi1^D51G^* ^/+^ and *Lgi1^D51G^* ^/*D51G*^ mice at P24. Scale bar, 100μm. (E) Immunofluorescence staining of Lgi1 in the cortices of wild‐type, *Lgi1^D51G^* ^/+^ and *Lgi1^D51G^* ^/*D51G*^mice at P24. Scale bar, 20μm. (F‐H) The expression (G) and secretion (H) of Lgi1 protein in cortical neurons extracted from wild‐type (secretion, 100.0 ± 8.8%, *n* = 5), *Lgi1^D51G^* ^/+^(secretion, 45.7 ± 13.9%, *n* = 5) and *Lgi1^D51G^* ^/*D51G*^ (secretion, 32.1 ± 5.6%, *n* = 4) mice. Data are shown as mean ±SEM. **p < 0.01; ns, not significant. One‐way ANOVA followed by Tukey's multiple comparisons test

Next, we explored the pathomechanism of D51G mutation in *Lgi1^D51G^* mutant mice. We found that the Lgi1 protein was reduced in the cerebral cortex of *Lgi1^D51G^* ^/+^ and *Lgi1^D51G^* ^/^ *^D51G^* mice to approximately 49% and 17% of that in the wild‐type mice, respectively (Figure 3A,B); however, the mRNA level remained unchanged (Figure 3C). Additionally, immunohistochemical analysis showed that the wild‐type Lgi1 protein was expressed mainly in the neuropil of the hippocampus. In contrast, the D51G mutant protein was excluded from the neuropil and instead accumulated in the cell body layers of the *Lgi1^D51G^* ^/+^ and *Lgi1^D51G^* ^/^ *^D51G^* mice (Figure 3D). Immunofluorescence analysis demonstrated the distribution of Lgi1 protein out of the cell bodies in the cortex of the wild‐type mice but not the *Lgi1^D51G^* ^/^ *^D51G^* mice. *Lgi1^D51G^* ^/+^ mice displayed partial Lgi1 secretion (Figure 3E).

To further verify the secretion defect of Lgi1^D51G^, we measured the expression and secretion of Lgi1 protein in cultured cortical neurons of wild‐type, *Lgi1^D51G^* ^/+^ and *Lgi1^D51G^* ^/^ *^D51G^* mice. While the Lgi1 protein was only modestly reduced in the cell lysis of *Lgi1^D51G^* ^/+^ and *Lgi1^D51G^* ^/^ *^D51G^* cultures, the Lgi1 secretion was significantly reduced in both *Lgi1^D51G^* ^/+^ and *Lgi1^D51G^* ^/^ *^D51G^* cultures to about 46% and 32% of that in the wild‐type culture, respectively (Figure 3F‐H). Thus, these results demonstrate that the secretion of Lgi1^D51G^ protein is disrupted in neurons, thus explaining the pathomechanism of *Lgi1^D51G^* mutation.

### 3.5 Antiepileptic drugs elongate the life of *Lgi1^D51G^* ^/^ *^D51G^* mice

**FIGURE 4:**
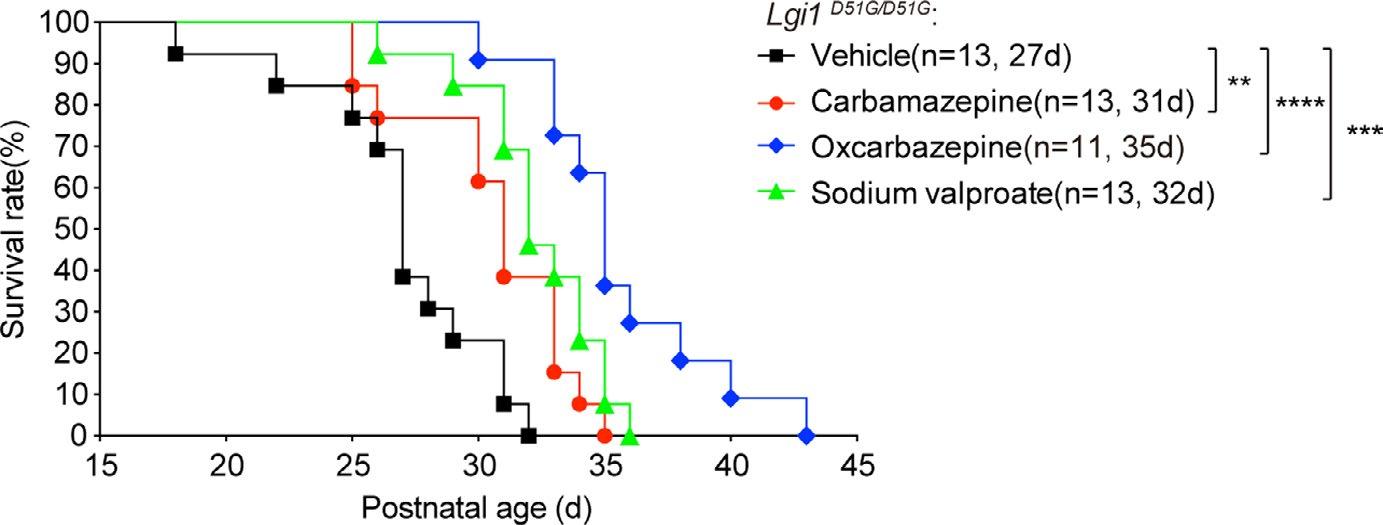
Antiepileptic drugs elongate the life of *Lgi1^D51G^* ^/*D51G*^ mice. Kaplan‐Meier survival plots of *Lgi1^D51G^* ^/*D51G*^ mice administered with vehicle (*n* = 13), carbamazepine (*n* = 13), oxcarbazepine (*n* = 11) and sodium valproate (*n* = 13). The median survival was indicated. **p < 0.01; *** *p* < 0.001****p < 0.0001; log‐rank test

The aforementioned observations demonstrate that the *Lgi1^D51G^* knock‐in mice are valid models for understanding the pathomechanism of LGI1^D51G^‐induced human ADLTE. Thus, we tested the effects of the commonly used antiepileptic drugs, carbamazepine, oxcarbazepine, and sodium valproate, on *Lgi1^D51G^* ^/^ *^D51G^* mice. The drugs were added to the drinking water and administered to the mother mice 14‐day after delivery and to their pups after weaning. The survival curves were monitored. The median survival time of the vehicle‐treated *Lgi1^D51G^* ^/^ *^D51G^* mice was 27 days. After treatment with carbamazepine (*n* = 13), oxcarbazepine (*n* = 11), or sodium valproate (*n* = 13), the median survival time was 31, 35, and 32 days, respectively (Figure 4A). Thus, all three drugs effectively prolonged the survival time of the *Lgi1 ^D51G^* ^/^ *^D51G^* mice, indicating that they can be effective in treating patients with *LGI1* mutations. Furthermore, these preliminary observations suggest that oxcarbazepine is slightly more effective than carbamazepine and sodium valproate.

## 4 DISCUSSION

In this study, we identified a novel c.152A>G; p. D51G mutation in exon 1 of the *LGI1* gene in a Chinese family with ADLTE. Patients with D51G heterozygous mutation of *LGI1* experienced partial seizures, which were effectively treated with carbamazepine. Although the mutation was also detected in two asymptomatic individuals of the family, which suggested incomplete penetrance, majority of the mutation carriers were symptomatic, indicating that the mutation is a high‐risk factor. The causality between the mutation and the symptoms was further confirmed using genetically modified *Lgi1^D51G^* mice. Thus, the detection of the *LGI1^D51G^* mutation is invaluable for individuals in this family with ADLTE, as it will help them seek genetic diagnosis prior to the onset of the symptoms. Furthermore, this information provides reliable genetic information for prenatal diagnosis.

Epilepsy is a common neurological disorder with a complex pathogenesis that is not yet fully understood. Recent studies indicate that dysfunction of the neurovascular activity, neuroinflammation, and metabolic state of the brain may contribute to epilepsy symptoms.^37^, ^38^, ^39^, ^40^, ^41^ Furthermore, next‐generation sequencing studies have revealed an increasing number of inherited gene mutations relevant to epilepsy,^42^, ^43^ which would help improve the diagnosis and develop personalized treatments for the patients and their family.

Up to 50% of families with ADLTE show inherited gene mutations of *LGI1*.^6^, ^7^ LGI1 is a secretory neuronal protein composed of N‐terminal LRR domain and C‐terminal epitempin repeat (EPTP) (also called EAR) domain.^9^, ^14^, ^44^ The “D” at position 51 is located within the N‐terminus of the LRR domain (LRRNT). To date, five disease‐causing mutations, C42R, C42G, C46R, C46F, and P43R, have been identified at LRRNT. The P43R mutation is likely to cause improper folding of the protein.^45^ The other four mutations disrupt intramolecular disulfide bonds, which may be critical in stabilizing the N‐terminal edges of LRR.^9^ Several secretion‐defective mutations have been reported to affect the structure of LGI1. For example, the E383A mutation destabilizes the β‐propeller structure of the EPTP domain of LGI1, which is recognized by the endoplasmic reticulum (ER) quality‐control system. Misfolded *LGI1* ^E383A^ is retained in the ER, and it subsequently enters the ER‐associated degradation pathway.^8^ In this study, we found that the Lgi1 protein was significantly reduced in both *Lgi1^D51G^* ^/+^ and *Lgi1^D51G^* ^/^ *^D51G^* mice but remained unaltered at the mRNA level. Indeed, immunohistochemistry analysis of the brain slices and secretion analysis of cortical neuronal cultures indicated that the protein secretion was defective in *Lgi1^D51G^* ^/^ *^D51G^* mice. It is possible that the substitution of the negatively charged “D” with the smallest “G” at LRRNT disrupts protein conformation. Subsequently, the misfolded protein is degraded, which in turn hampers the physiological functions of LGI1 and causes an epileptic phenotype.

To date, over 40 *LGI1* mutations have been identified in ADLTE patients.^8^, ^9^, ^10^ The pathogenesis of these mutations has mostly been analyzed in recombinant systems.^10^ Majority of these mutations disrupt LGI1 secretion, while some affect LGI1 binding to its ADAM22 or ADAM23 receptor, and a few impair LGI1 dimerization.^9^ Animal models have been developed to characterize the pathophysiology of these mutants in vivo. Overexpression of the P43R mutation in the *Lgi1* knockdown zebrafish was used to evaluate its effects on animal motion.^45^ Mouse models of familial epilepsy have been recently developed using a transgenic strategy, which re‐expresses the transgenic mutant *Lgi1* in homozygous knockout mice *(Lgi1* ^−/−;^ *^mutation^*).^8^, ^27^ Transgenic mice are not only valuable for understanding the pathomechanisms of the mutations, but also provide a useful tool to screen for personalized treatments. Remarkably, the seizure susceptibility of mice carrying the transgenic E383A mutation, which caused Lgi1 degradation in the ER, was effectively relieved by 4PBA, a small molecule correcting misfolded proteins in the ER. However, drawbacks arguably exist with the transgenic strategy, such as unknown gene integration sites and numbers and genetic instability, and thus unusual gene expression levels. Recent developments in genome engineering tools, such as the powerful CRISPR‐Cas9 system, allow precise editing of the mouse genome at specific loci.^34^, ^35^ Therefore, we generated *Lgi1^D51G^* knock‐in mice by mutating a single nucleotide at exon 1 of the *Lgi1* gene (Figure 2A). This is superior to the transgenic models because the gene expression level of the mutant *Lgi1* is tightly controlled by endogenous promoters and/or other regulatory elements. As expected, the level of *Lgi1* mRNA in the mutant mice was the same as that in wild‐type mice (Figure 3C). EEG recordings in *Lgi1^D51G^* ^/+^ mice displayed spontaneous epileptiform discharges in approximately half of the mice in 1 week, consistent with the incomplete penetrating phenotype observed in human patients. Therefore, *Lgi1^D51G^* ^/^ *^D51G^* and *Lgi1^D51G^* ^/+^ mice are valid models for investigating the pathomechanism of *LGI1* ^D51G^ mutation‐induced human ADLTE and for exploring personalized treatments. We administered the antiepileptic drugs, carbamazepine, oxcarbazepine, and sodium valproate, in *Lgi1^D51G^* ^/^ *^D51G^* mice and found that all drugs increased the survival time of the mice; oxcarbazepine appeared to be more effective than carbamazepine and sodium valproate. The effects of antiepileptic drugs on *LGI^D51G^* ^/+^ patients need to be verified in future studies.

## CONFLICTS OF INTEREST

None declared.

## AUTHOR CONTRIBUTIONS

Ping Hu, Yan Wang, Fengchang Qiao, and Zhengfeng Xu collected the data from patients; Dan Wu designed the knock‐in mouse model; Dan Wu, Xiao‐Yu Teng, Qing‐Qing Li, and Tingting Liu analyzed Lgi1 expression; Yan‐Yu Zang, Ya‐Ping Zhou, and Qi‐Gang Zhou performed EEG recordings; Yan‐Yu Zang, Jiang Chen, Jia‐Hui Sun, and Hao‐Yang Feng monitored spontaneous and induced epileptic behaviors; Yun Stone Shi and Zhengfeng Xu supervised the study; and Yun Stone Shi and Dan Wu wrote the paper.

## Supporting information

Supplementary Material

Video S1

## Data Availability

The data that support the findings of this study will be available from the corresponding author upon reasonable request.
