# Supplementary material for: A novel LGI1 mutation causing autosomal dominant lateral temporal lobe epilepsy confirmed by a precise knock‐in mouse model
Source: CNS Neurosci Ther. 2021 Nov 12;28(2):237–46. doi: 10.1111/cns.13761 (PMC8739050; doi:10.1111/cns.13761)

Full unedited blot for Figure 3A

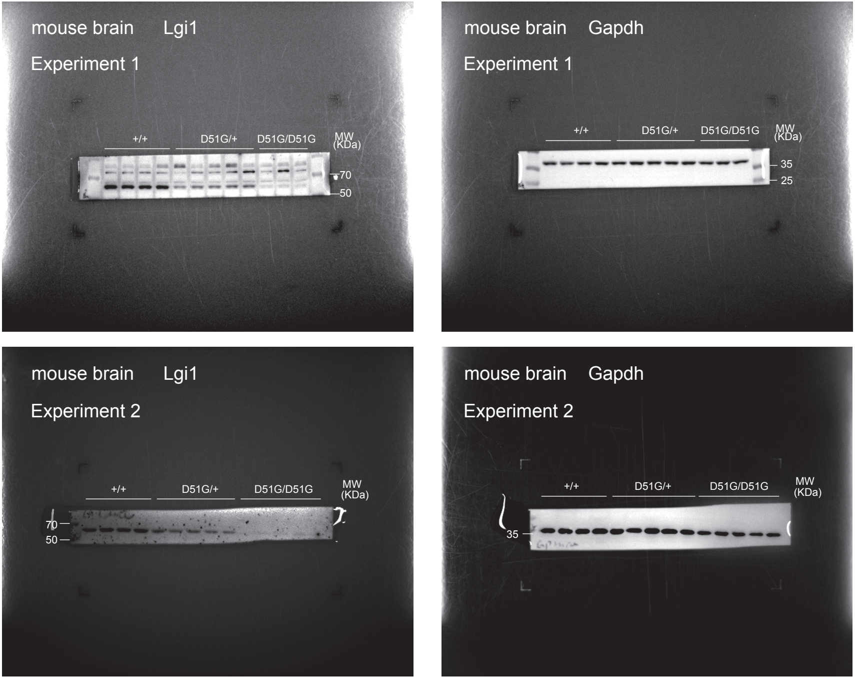

Full unedited blot for Figure 3F

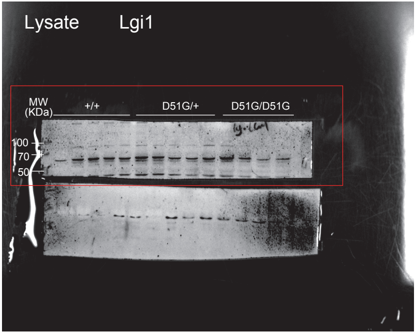

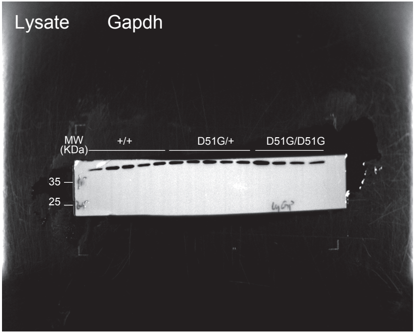

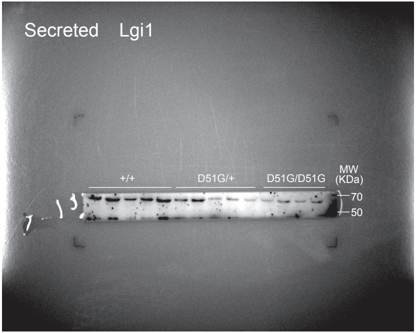

Supplement: Supplementary file 1 — Supplementary Material [file CNS-28-237-s002.pdf]
